# Characterizations of Water-Soluble Chitosan/Curdlan Edible Coatings and the Inhibitory Effect on Postharvest Pathogenic Fungi

**DOI:** 10.3390/foods13030441

**Published:** 2024-01-30

**Authors:** Youwei Yu, Kunyu Liu, Shaoying Zhang, Liangliang Zhang, Jiaqi Chang, Ziyu Jing

**Affiliations:** College of Food Science, Shanxi Normal University, Taiyuan 030031, China; 222420005@sxnu.edu.cn (K.L.); 324139@sxnu.edu.cn (S.Z.); 703581@sxnu.edu.cn (L.Z.); 2030010129@sxnu.edu.cn (J.C.); 2030010132@sxnu.edu.cn (Z.J.)

**Keywords:** chitosan, curdlan, edible coating, preservation

## Abstract

This study focused on developing a composite coating comprising water-soluble chitosan (CTS) and curdlan (CUR). Cherry tomatoes served as the test material for assessing the preservative efficacy of these coatings. The incorporation of CUR markedly enhanced the coating’s surface properties, refined its molecular structure, and improved its tensile strength and elongation at break. Additionally, the coating demonstrated enhanced permeability to water vapor, oxygen, and carbon dioxide and improved light transmission. The storage experiment, conducted at 25 ± 1 °C with a relative humidity of approximately 92% over 10 days, revealed that the CTS/CUR composite coating at a 1:1 ratio significantly outperformed the individual CTS or CUR coating and uncoated samples in maintaining the quality of postharvest cherry tomatoes. The 1:1 CTS/CUR composite coating demonstrated superior preservative effects. This study suggested that water-soluble chitosan/curdlan composite coatings have considerable potential for use in the preservation of postharvest fruits and vegetables.

## 1. Introduction

Edible coating technology is considered an effective method for fruit and vegetable preservation [1,2]. Currently, natural polysaccharides have become a research focus, due to their food safety and degradability. However, the preservation effect of pure coating is often not ideal. Composite coatings can compensate for the deficiencies of pure coatings, offering broader application prospects [3]. Chitosan (CTS) is a natural multifunctional biopolymer [4] that is abundant in the cell membranes of marine arthropods, fungi, insects, algae, and cell walls of higher plants. Water-soluble chitosan, derived from chemical modification, exhibits good antifungal activity and barrier properties against water vapor and oxygen. As a result, the use of water-soluble chitosan in food packaging has increased in recent years. Curdlan (CUR), a linear structure composed of D-glucose through β-1,3-glucoside bonds [4], is a new type of extracellular polysaccharide. It possesses a triple helix molecular structure, with intramolecular and intermolecular hydrogen bonds. Due to its unique thermal gel and rheological properties [5], CUR is widely used in the food industry.

Both the pure water-soluble chitosan coating and pure curdlan coating exhibit poor barrier properties and water resistance. However, when the two polysaccharides interact through hydrogen bonds, they form a compact coating that enhances their properties [6]. Moreover, curdlan in water has a unique thickening ability, which could improve the properties of water-soluble chitosan/curdlan composite coatings [5,7]. Notably, curdlan and modified water-soluble chitosan were chosen as materials not commonly employed in preservation fields. The development of this new composite coating could be achieved through sustainable production, making it a potential environmentally friendly material for fruit preservation.

In this study, CTS and CUR were used to prepare edible CTS/CUR coatings with different ratios. The structure and properties of these coatings were comprehensively analyzed, including molecular interactions, morphology, water barrier properties, gas barrier properties, mechanical properties, and thermal stability. Composite coatings with varying proportions were applied to cherry tomatoes to assess their effectiveness in preventing *Botrytis cinerea* and *Alternaria* (Nees: Fr) infections during the preservation process. This study aimed to contribute to advances in research on CTS/CUR composite materials and to the progress of the field of fruit preservation.

## 2. Materials and Methods

### 2.1. Materials

Water-soluble chitosan (chitosan amino salt-forming type, generally lactate; molecular formula: (C_9_H_17_NO_7_)_n_) was obtained from Ao Kang Biotechnology Co., Ltd. Jinan, China). Curdlan was obtained from Green City Biology Co., Ltd. (Guangzhou, China). Glycerol was purchased from Fengchuan Chemical Reagent Technology Co., Ltd. (Tianjin, China). Cherry tomatoes were obtained from local markets (Taiyuan, China), and the fruits were bright in color and not mechanically damaged.

### 2.2. Preparation of Coatings

The coatings were prepared according to previous methods [5]. At 60 °C, the solid powders of CTS and CUR were mixed in specific proportions with deionized water. The total solid content was maintained at 1% (*w*/*v*), and the specific ratios were as follows: CTS:CUR = 1:0, 0:1, 1:0.5, 1:1, 1:1.5, 1:2, 0.5:1, 1.5:1, and 2:1 (*w*/*w*). A total of 0.1 mL of glycerol was added to 100 mL of the coating solution as a plasticizer. The mixture was stirred using a magnetic stirring pot (SHJ-6A, Changzhou Jintan Liangyou Instrument Co., Ltd., Changzhou, China) at 2000 rpm for 40 min until there were no particles in the mixture. Then, the mixture was degassed using a vacuum pump (2XZ, Zhejiang Huangyan Tianlong Vacuum Pump Co., Ltd., Taizhou, China) to remove any bubbles in the solution. Afterward, the solution was left to stand for 8 h. Then, 35 mL of the solution was cast on a 10 cm × 10 cm acrylic film and dried at 30 °C to form a film. The films were uncovered and stored in a vacuum pump at 25 ± 1 °C and approximately 68% relative humidity. If the storage time exceeded 24 h, all the films were removed.

### 2.3. Structural Characterization

#### 2.3.1. Fourier-Transform Infrared Spectroscopy (FTIR)

The microstructure and molecular interactions of the films were analyzed using Fourier-transform infrared spectroscopy (Nicolet iS50, Thermo Fisher Scientific Shier Technology Co., Ltd., Waltham, MA, USA). The horizontal ATR was in the range of 4000–400 cm^−1^, and infrared spectra were collected at a resolution of 0.25 cm^−1^.

#### 2.3.2. X-ray Diffraction (XRD)

The XRD patterns of the films were obtained using an X-ray diffractometer (Ultima IV-185, Rigaku Co., Ltd., Tokyo, Japan). The samples were irradiated by Cu Kα radiation for 4°/min at 40 kV and 40 mA, and the XRD images were recorded in the range of 2θ from 5° to 40°.

#### 2.3.3. Scanning Electron Microscopy (SEM)

The microstructure of the films was observed using scanning electron microscopy (JSM-7500F, JEOL, Ltd., Tokyo, Japan). The films were broken after freezing in liquid nitrogen, and the obtained films were sputtered with gold under vacuum. The surfaces of the films were observed at an accelerating voltage of 15 kV.

#### 2.3.4. Differential Scanning Calorimetry (DSC)

Differential scanning calorimetry (DSC; 200 F3, NETZSCH, Selb, Germany) was used to analyze the films. The thermal characteristics were analyzed using a nitrogen flow of 25 mL/min and a heating rate of 15 °C/min in the temperature range of 20 to 250 °C.

#### 2.3.5. Thermogravimetric Analysis (TGA)

Thermogravimetric analysis of the films was carried out using a thermogravimetric analyzer (TGA/DSC1/1600HT, METTLER-TOLEDO Co., Ltd., Zurich, Switzerland). The films (2 mg) were loaded into ceramic crucibles and heated at a rate of 10 °C /min from 25 °C to 700 °C under a N_2_ atmosphere (100 mL/min).

### 2.4. Physical Property Determination

#### 2.4.1. Moisture Content (MC)

The prepared films were subjected to constant conditions of 80% RH and 22 °C. Subsequently, the plants were dried in an oven (DHG-9030A, Yiheng Scientific Instrument Co., Ltd., Shanghai, China) at 105 °C until a constant weight was achieved. The water content of the films was calculated using the following formula:

$$MC\left(\%\right)=\frac{M_{0}-M_{t}}{M_{0}}\times 100\%$$

In the formula, *M*_0_ is the balanced weight of the sample, g; and *M_t_* is the final weight after drying, g.

#### 2.4.2. Water Solubility (WS)

The films were subjected to conditions similar to those of *MC*. The samples were then immersed in distilled water and left to stand at 22 °C for 24 h. Afterward, the films were stirred for 2 h. The coatings were removed and dried to a constant weight at 105 °C. The water solubility (*WS*) of the film was calculated as follows:

$$WS\left(\%\right)=\frac{M_{0}-M_{t}}{M_{0}}\times 100\%$$

where *M*_0_ is the initial weight of the film after equilibration, g; *M*_t_ is the final dry weight of the film after soaking, g.

### 2.5. Determination of Mechanical Properties

#### 2.5.1. Film Thickness

The thickness of the prepared films was determined using a gauge (YHT 103980, Yuanhengtong Technology Co., Ltd., Shenzhen, China). Ten points on different sites of the films were selected, ensuring that the center point was included. The film thickness was calculated as the average value of these ten points.

#### 2.5.2. Tensile Strength (TS) and Elongation at Break (EAB)

The tensile strength (TS) and elongation at break (EAB) of the films were measured using an intelligent electronic mechanics testing machine (5944, Instron Co., Ltd., Norwood, MA, USA). A strip with dimensions of 80 mm × 20 mm (l × b) was tested at a speed of 48 mm /min, and the maximum load and elongation length were recorded. The values of tensile strength (*TS*) and elongation at break (*EAB*) were calculated using the following formulas:

$$TS=\frac{F}{A}$$

$$EAB\left(\%\right)=\frac{L-L_{0}}{L_{0}}\times 100\%$$

In the formula, *F* is the maximum force, n; *A* is the cross-sectional area, mm^2^; *L* is the length of the film when it breaks, mm; and *L*_0_ is the initial length of the film, mm.

### 2.6. Determination of Barrier Performance

#### 2.6.1. Light-Blocking Performance and Transparency

The light-blocking performance was determined according to a previously established method [8]. The films were cut into strips that could adhere to the inner wall of a vertical cuvette, ensuring that there were no bubbles present on the inner wall. As a blank control, the light absorption values of the films were recorded in the wavelength range of 400~800 nm [9], and the light absorption value at 600 nm was considered the transparency value [10]. The light transmittance (*T*) and transparency (*T*_0_) were calculated using the following formulas:

$$T=0.1^{A}\times 100\%$$

$$T_{0}=-\frac{\log T600}{d}$$

In the formula, *A* is the absorption at different wavelengths; *T* is the light transmittance; *T*_0_ is the transparency; T600 is the transmittance of the film at 600 nm; and d is the average thickness of the coating, mm.

#### 2.6.2. Water Vapor Transmission Rate (WVTR)

The water vapor transmission rate was assessed according to previous methods [11]. A conical bottle was filled with no more than 2/3 distilled water. The mouth of the conical bottle was securely wrapped and sealed with prepared films. The conical bottle was subsequently placed in an oven at 30 °C and with a relative humidity of 13 ± 2%. The mass of the conical bottle was weighed every 12 h and measured three times, consecutively. The water vapor permeability was calculated using the following formula:

$$WVTR=\frac{\Delta m}{A\times T}$$

In the formula, *WVTR* is the steam permeability of water, g/(h·m^2^); Δ*m* is the weight bottle mass loss, g; *A* is the area of the membrane, m^2^; and *T* is the determination time, h.

#### 2.6.3. Carbon Dioxide Permeability Measurement (CDP)

CO_2_ permeability was determined in accordance with the method outlined in GB/T 1038–2022 [12]. The CO_2_ concentration was determined using a carbon dioxide gas analyzer (F-940, Sunshine Yishida Technology Co., Ltd., Beijing, China). The CO_2_ permeability was calculated using the following formula:

$$CDP=\frac{V_{CO_{2}}\times d}{A\times \Delta P}$$

In the formula, *CDP* is the CO_2_ permeability, cm^2^/(*d*·kPa); *A* is the effective air-permeable area of the membrane, cm^2^; d is the average thickness of the membrane, cm; Δ*P* is the difference in gas pressure between the two sides of the membrane during measurement; and *V*co_2_ is the stable carbon dioxide permeable gas volume at 24 h, mL. 

#### 2.6.4. Oxygen Barrier Property

Oxygen barrier property was measured by sodium thiosulfate titration [13]. First, 3 g of soybean oil was placed into 50 mL conical bottles of the same size. These conical bottles were then sealed with different composite membranes. One bottle without a membrane seal was designated as the blank group. All the films were subsequently placed in an incubator at 50 °C for 3 days, after which the peroxide value (PV) was measured. The peroxide value of the oil was determined in accordance with GB 5009.227. The PV was calculated using the following formula:

$$PV=\frac{\left(V-V_{0}\right)\times c\times 0.1269}{m}\times 100$$

In the formula, *PV* is the peroxide value, g/100 g; *V* is the volume of sodium thiosulfate standard solution consumed by the sample, mL; *V*_0_ is the volume of sodium thiosulfate standard solution consumed in the blank sample, mL; c is the concentration of sodium thiosulfate standard solution, mol/L; 0.1269 is the mass of iodine equivalent to 1.00 mL of standard titration solution of sodium thiosulfate, [c(Na_2_S_2_O_3_)] = 1.000 mol/L; *m* is the sample mass, g; and 100 is the conversion factor.

### 2.7. Antifungal Activity Determination

In the process of preparing PDA, different membrane solutions were added to the PDA culture. *Botrytis cinerea* and *Alternaria* (Nees: Fr), cultivated for 10 days, were punched using a 12 mm diameter hole punch to obtain a cake. The two fungal strains were subsequently cultured in a constant temperature incubator at 26 ± 1 °C for 4 days. The colony diameter was measured every 2 days using the cross method.

### 2.8. Preservation Experiment of Cherry Tomatoes

Cherry tomatoes were washed with distilled water and soaked in a coating solution for 30 min. Then, aseptically, a 2 mm diameter hole was made in each of the 115 cherry tomatoes. Next, 20 μL of fungal suspension (10^6^) was injected into the hole of each fruit and allowed to dry at room temperature. Finally, the fruits were stored at a temperature of 25 ± 1 °C and a relative humidity of approximately 92% for 10 days.

### 2.9. Statistical Analysis

All assays were repeated in triplicate. The experimental data were statistically analyzed using SPSS statistics software (Statistcs17.0, IBM, Ltd., Armonk, NY, USA) and compared among groups using one-way analysis of variance (ANOVA) with the Duncan multiple range test (*p* ≤ 0.05). Graphs were generated using Origin software (Origin2023b, OriginLab, Ltd., Northampton, MA, USA).

## 3. Results

### 3.1. Structural Characterization

#### 3.1.1. FTIR

**Figure 1:**
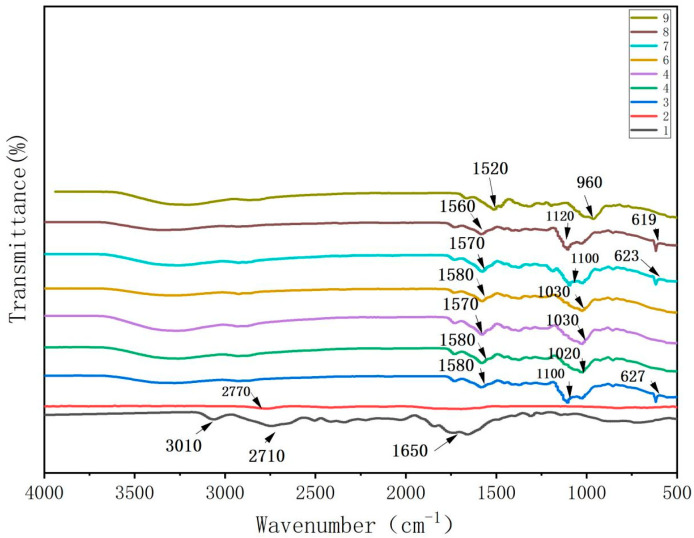
FTIR spectrum of water-soluble chitosan (CTS)/curdlan (CUR) films. In this figure, 1, 2, 3, 4, 5, 6, 7, 8, and 9 replaced CTS:CUR (*w*/*w*) = 1:0, 0:1, 1:0.5, 1:1, 1:1.5, 1:2, 0.5:1, 1.5:1, and 2:1, respectively.

As shown in Figure 1, the absorption peaks observed in the FTIR spectra of the CTS films were attributed to the stretching vibrations of -CHN at 3010 cm^−1^, -CH2 at 2710 cm^−1^, and C-O at 1650 cm^−1^ [14]. The weak peak at 2770 cm^−1^ in the spectrum of the CUR film was attributed to C-H stretching vibrations. The peak at 1620 cm^−1^ represented the C-O stretching vibration [15], while the broad peak at 822 cm^−1^ indicated -NH2 bending.

In the FTIR spectra of the CTS/CUR composite films with different proportions of the plasticizer glycerol, the peaks in the range of 1520~1580 cm^−1^ were attributed to the bending and stretching vibrations of the amino groups and their protonated counterparts. The peaks in the range of 960~1120 cm^−1^ were associated with the interaction between the film structure and the -OH group of glycerol, confirming the presence of glycosidic bonds. The absorption peak at approximately 620 cm^−1^ was due to the out-of-plane bending vibration of C-H. Overall, the peak value remained relatively unchanged for the different proportions of the CTS/CUR composite films. Compared to those of the pure CTS and CUR films, the peaks at 3080 cm^−1^ and 2750 cm^−1^ disappeared in the spectrum of the composite films, while new peaks emerged. This indicated potential intermolecular hydrogen bonding interactions between CTS and CUR and between CUR and glycerol.

#### 3.1.2. XRD

**Figure 2:**
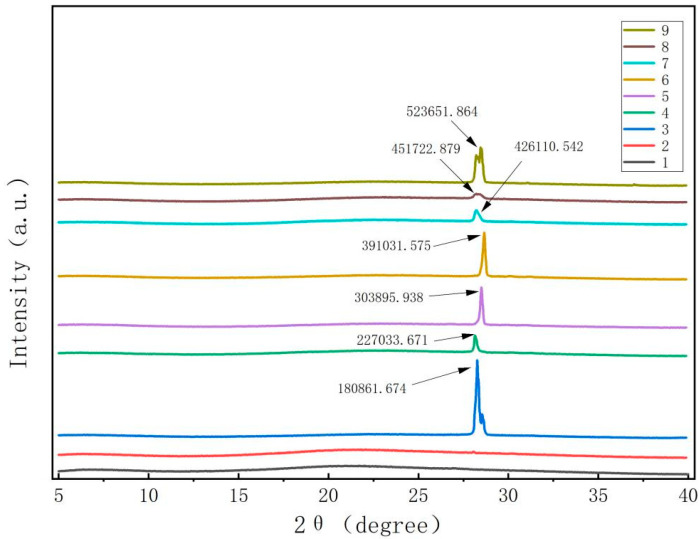
XRD patterns of water-soluble chitosan (CTS)/curdlan (CUR) films. In this figure, 1, 2, 3, 4, 5, 6, 7, 8, and 9 replaced CTS:CUR (*w*/*w*) = 1:0, 0:1, 1:0.5, 1:1, 1:1.5, 1:2, 0.5:1, 1.5:1, and 2:1, respectively.

Figure 2 demonstrates that the pure films of CTS and CUR did not exhibit any apparent X-ray diffraction peaks. However, once the composite films were prepared by adding CUR and glycerol, the samples exhibited a sharp and prominent absorption diffraction peak at 2θ = 28.1°. Although the heights and widths of the peaks differed among the various composite films, the differences were minimal. These results indicate that the polysaccharide molecules of CTS and CUR possess notable film-forming crystallinity [16]. Notably, when the CTS:CUR (*w*/*w*) = 1:0.5, the intensity of the characteristic peak was strong. Conversely, when the CTS:CUR (*w*/*w*) = 0.5:1, the intensity of the characteristic peak noticeably weakened. This difference may be attributed to the interaction between CTS and CUR, which disrupts the natural crystal structure of both CTS and CUR, consequently leading to a decrease in crystallinity.

#### 3.1.3. SEM

**Figure 3:**
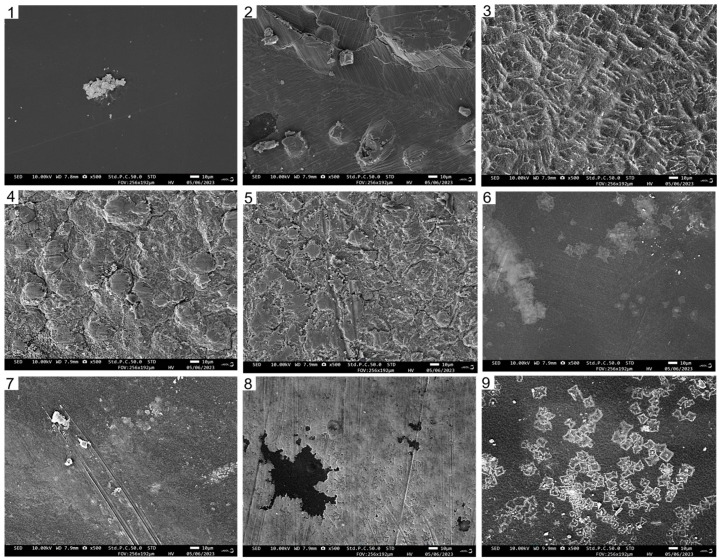
The scanning electron microscope photos of the surfaces of water-soluble chitosan (CTS)/curdlan (CUR) films. In this figure, **1**, **2**, **3**, **4**, **5**, **6**, **7**, **8,** and **9** replaced CTS:CUR (*w*/*w*) = 1:0, 0:1, 1:0.5, 1:1, 1:1.5, 1:2, 0.5:1, 1.5:1, and 2:1, respectively.

As depicted in Figure 3, the surface of the pure CTS film appeared uniform and continuous, without any prominent wrinkles. Moreover, the CUR film exhibited fine cracks and protrusions. In the composite films, a higher content of CUR corresponded to a decreased density of surface wrinkles. Notably, when the CTS:CUR (*w*/*w*) ratio was 1:2, the composite film had the smoothest surface. This observation can likely be attributed to the formation of hydrogen bonds between CTS and CUR, which subsequently alters the original connection state of the polymer matrix.

#### 3.1.4. DSC

**Figure 4:**
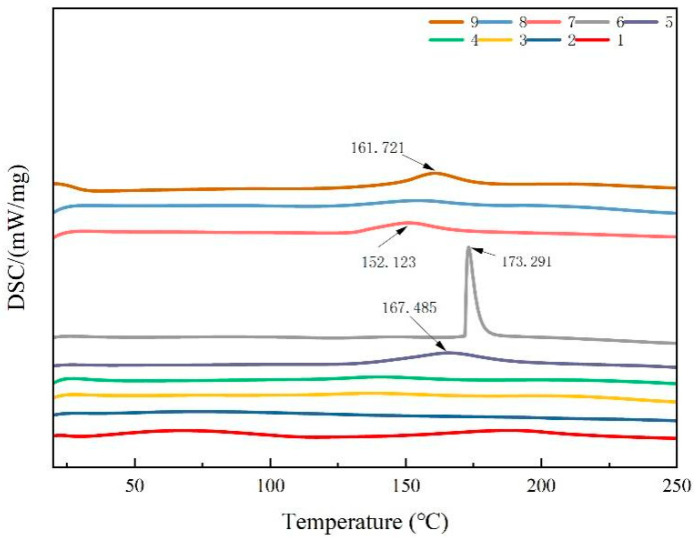
DSC thermograms of water-soluble chitosan (CTS)/curdlan (CUR) films. In this figure, 1, 2, 3, 4, 5, 6, 7, 8, and 9 replaced CTS:CUR *(w/w*) = 1:0, 0:1, 1:0.5, 1:1, 1:1.5, 1:2, 0.5:1, 1.5:1, and 2:1, respectively.

The thermal stability of the composite films was evaluated by comparing the peak endothermic temperatures. A higher peak temperature indicated a more stable structure. As illustrated in Figure 4, the DSC curves of the composite films displayed exothermic peaks at 152.1, 161.7, 167.4, and 173.2 °C. Among these, composite film No. 6 exhibited a larger thermal degradation peak, indicating that it had the highest thermal stability among the blended films. The thermal stability of the blend films was likely derived from their favorable miscibility. Furthermore, as the CTS content decreased in the composite films, there was no significant shift in the thermal degradation peak. However, compared to those of the pure CTS and CUR coatings, the intensity of the thermal degradation peak increased, indicating that the crystalline domains and hydrogen bonds between CTS and CUR interacted and consequently altered the thermal stability of the films.

#### 3.1.5. TGA

**Figure 5:**
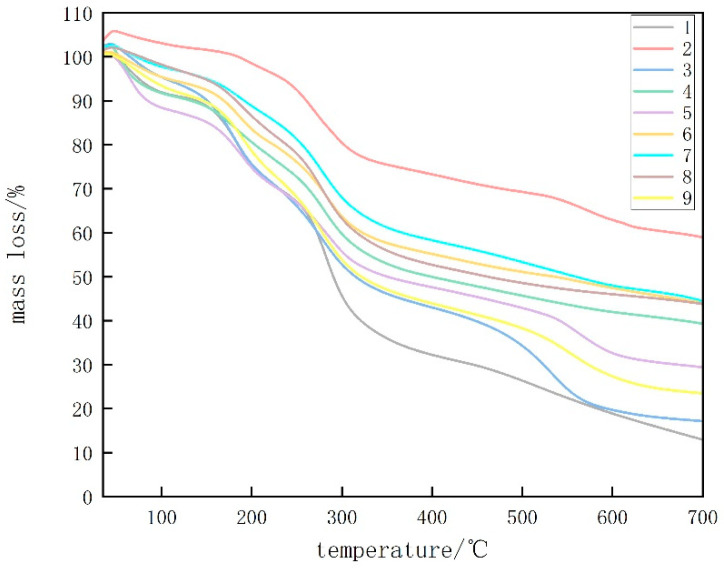
TGA thermograms of water-soluble chitosan (CTS)/curdlan (CUR) films. In this figure, 1, 2, 3, 4, 5, 6, 7, 8, and 9 replaced CTS:CUR (*w*/*w*) = 1:0, 0:1, 1:0.5, 1:1, 1:1.5, 1:2, 0.5:1, 1.5:1, and 2:1, respectively.

Figure 5 displays TGA analysis diagrams of CTS, CUR, and the composite membrane films with varying proportions. The degradation of glycerol was observed between 180 and 250 °C. According to Table 1, the highest temperature required for CUR removal was determined, and the highest temperature remaining at 700 °C was used. This can be attributed to the preparation of the previous film at 80 °C, which resulted in the formation of a stable, irreversible gel in the CUR film [17]. For the composite coating films with a CTS:CUR ratio of 0.5:1 (*w*/*w*), the highest temperature was required at mass losses of 10% and 25%. Additionally, a higher temperature was needed at a mass loss of 50%, which can be attributed to the favorable interactions between CTS and CUR and between CUR and glycerol at this ratio. Consequently, the thermal stability of the edible films improved [18]. The residue analysis at 700 °C indicated that the residue gradually decreased with increasing CTS content, reaching its lowest point at a CTS:CUR (*w*/*w*) ratio of 1:0.5. This highlights the good degradation performance of the edible film prepared at this ratio.

### 3.2. Structural Characterization

The transfer of water vapor both inside and outside the coating is a significant factor in food packaging [19]. As shown in Table 2, the MC, WS, and opacity gradually decreased with increasing concentrations of CUR. The addition of CUR reduced the number of molecular interactions within the films and increased the number of fine lines on the surface. WS, a factor influencing the biodegradability and water resistance of films [20], was affected by the addition of CUR to CTS film substrates. This can be explained by the cross-linking between the CTS component and the hydrophilic groups of CUR. The resulting cross-linking in the membrane matrix led to decreased water solubility, a membrane with low water affinity, and subsequently, decreases in water content, water solubility, and transparency. These properties contribute to the overall quality of the composite membrane.

### 3.3. Determination of Mechanical Properties

As depicted in Table 3, the thickness of the prepared films increased with increasing concentration of CUR. The presence of cracks and protrusions in the CUR structure may result in an increase in the spatial distance within the film matrix, thus contributing to the increase in thickness of the composite films. It is important for composite films to possess a certain level of TS and EAB to prevent cracking when applied to products [21]. The greater the TS and EAB of the edible films are, the better their ability to withstand external forces caused by environmental changes without experiencing internal cracking [22]. The mechanical characteristics of the films are closely related to the adhesion stability of the coating on fruits and vegetables. As outlined in Table 3, an increase in the concentration of CUR enhanced the elongation at break of the composite films but significantly reduced the tensile strength. The TS concentration exhibited an inverse relationship with the CUR concentration, while the EAB concentration increased with increasing CUR concentration. The TS and EAB data for the pure films indicated that the TS of the pure CUR film was superior to that of the pure CTS film. Among the composite films, the film with a mass ratio of CTS to CUR of 1:2 exhibited the highest elongation at break, suggesting that the addition of CUR to the CTS film can enhance its flexibility, although its tensile strength decreases. This difference may be attributed to the structural changes induced by the increase in CUR concentration, which led to improved TS. According to the SEM images, as the CUR concentration increased, the number of cracks and voids on the surface of the composite films also increased. Therefore, the variation in the mechanical properties may be attributed to the intermolecular interactions of the composite films.

### 3.4. Barrier Performance

#### 3.4.1. Analysis of Light-Blocking Performance

**Figure 6:**
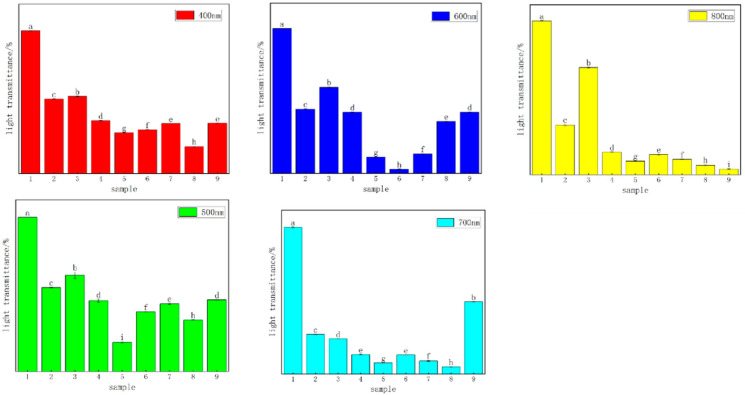
Results of light transmittance analysis of water-soluble chitosan (CTS)/curdlan (CUR) films. Means in each column with different superscript letters are significantly different (*p* ≤ 0.05). In this table, 1, 2, 3, 4, 5, 6, 7, 8, and 9 replaced CTS:CUR (*w*/*w*) = 1:0, 0:1, 1:0.5, 1:1, 1:1.5, 1:2, 0.5:1, 1.5:1, and 2:1, respectively.

The light transmittance reflects the degree of intermolecular compatibility. A higher light transmittance corresponds to stronger intermolecular compatibility. As shown in Figure 6, the light transmittance of the CTS pure film was the highest, while the light transmittance of the composite films slightly decreased. We hypothesized that there would be some differences in the properties of CTS and CUR. After the films were dried, their surfaces became rough, which affected the light transmittance of the films. The experimental results indicated that the addition of CUR influenced the physical and chemical properties of the CTS film, with certain properties improving while others tended to decrease. The light transmittance of the composite films decreased with increasing CUR concentration, with No. 6 exhibiting the lowest transmittance.

#### 3.4.2. Analysis of Gas Barrier Performance

The water permeability of the film is low, making it difficult for water to be lost from fruits and vegetables. This low water permeability is beneficial for preserving fruits and vegetables after harvest. Furthermore, a film with high O_2_ permeability and low CO_2_ permeability is advantageous for creating a micro-controlled atmosphere and inhibiting the reproduction and growth of aerobic microorganisms. The peroxide concentration is an indicator of the oxygen barrier properties of a film, with lower values indicating better oxygen barrier properties.

As presented in Table 4, the water permeability of the composite films decreased with increasing CUR concentration. This difference may be due to the alteration in the hydrophilicity of the CTS film, caused by the addition of CUR. The combination of CUR and CTS resulted in a change in the crystal structure of CTS, leading to the formation of a hydrophobic microporous structure in the composite films and reducing water permeability. Among all the films, the pure CUR film exhibited the lowest CO_2_ permeability, while the pure CTS film had the highest CO_2_ permeability. Among the composite films, the CO_2_ permeability was lowest when the proportion of CTS:CUR (*w*/*w*) was 2:1. This finding suggested that CTS has better CO_2_ permeability than CUR. The transmission rate of O_2_ is determined by the peroxide value, with higher values indicating a higher degree of oil oxidation and a higher rate of O_2_ transmission through the films. As shown in Table 4, the peroxide concentration increased with increasing CUR concentration, reaching the highest value at sample 6.

### 3.5. Antifungal Activity

**Figure 7:**
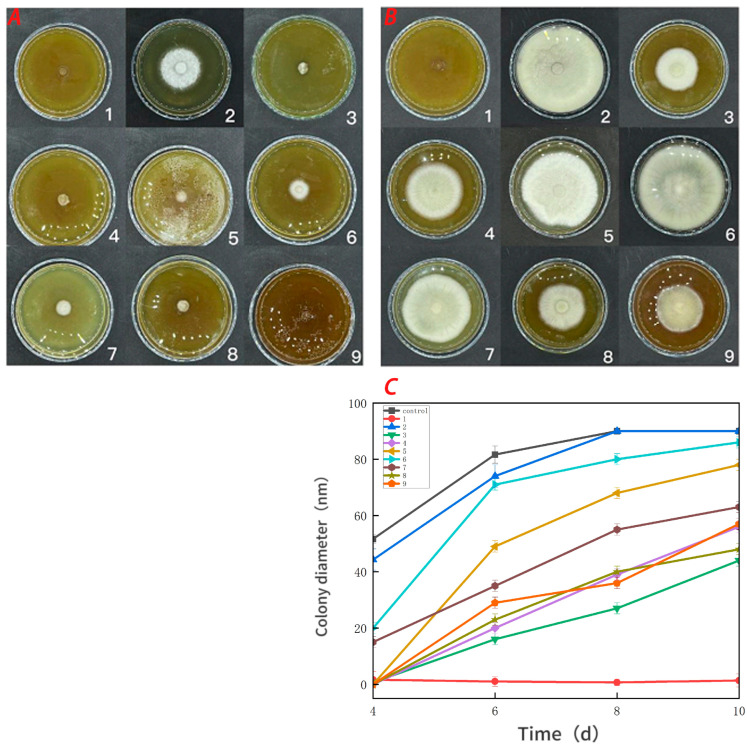
Effect of water-soluble chitosan (CTS)/curdlan (CUR) coatings on the growth of *Botrytis cinerea (***C**) on PDA media; colony diameter was measured after 4 days of incubation at 26 °C, and photos were taken on the fourth day (**A**) and the tenth day (**B**). Bars represents the standard error of the average. Different letters indicate significant differences (*p* < 0.05). In this figure, 1, 2, 3, 4, 5, 6, 7, 8, and 9 replaced CTS:CUR (*w*/*w*) = 1:0, 0:1, 1:0.5, 1:1, 1:1.5, 1:2, 0.5:1, 1.5:1, and 2:1, respectively.

**Figure 8:**
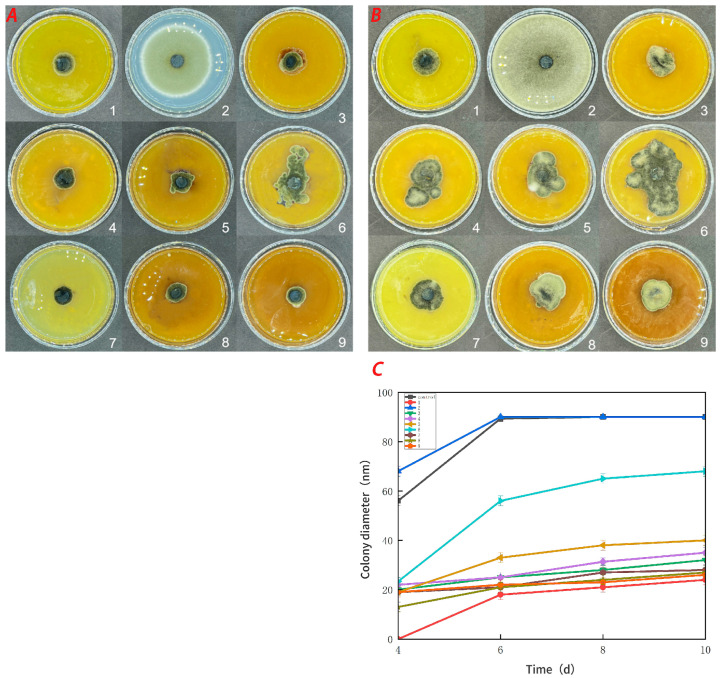
Effect of water-soluble chitosan (CTS)/curdlan (CUR) coatings on the growth of *Alternaria* (Nees: Fr) (**C**) on PDA media; colony diameter was measured after 4 days of incubation at 26 °C, and photos were taken on the fourth day (**A**) and the tenth day (**B**). Bars represents the standard error of the average. Different letters indicate significant differences (*p* < 0.05). In this figure, 1, 2, 3, 4, 5, 6, 7, 8, and 9 replaced CTS:CUR (*w*/*w*) = 1:0, 0:1, 1:0.5, 1:1, 1:1.5, 1:2, 0.5:1, 1.5:1, and 2:1, respectively.

Figure 7 demonstrates that the pure CTS coating strongly inhibited *Botrytis cinerea*. CUR had a weaker inhibitory effect on *Botrytis cinerea*, but it was more effective than the control treatment. With an increase in the CUR concentration, the CTS/CUR composite coatings demonstrated a certain inhibitory effect on *Botrytis cinerea*, although the inhibitory effect became weaker. Figure 8 shows the inhibitory effect on *Alternaria* (Nees: Fr). The inhibitory effect of the pure CTS coating on *Alternaria* (Nees: Fr) was weaker than that on *Botrytis cinerea*. The inhibitory effect of CUR on *Botrytis cinerea* was very weak, and it may even promote the growth of *Alternaria* (Nees: Fr). However, in the composite coating treatment, the inhibitory effect on *Alternaria* (Nees: Fr) was significantly greater than that on *Botrytis cinerea*. These antifungal experimental results are consistent with those of previous studies [23] and demonstrate that, compared with CUR, CTS has a stronger inhibitory effect on fungi. These antifungal effects align with the aforementioned analysis of barrier performance.

### 3.6. Preservation Experiment of Cherry Tomatoes

**Figure 9:**
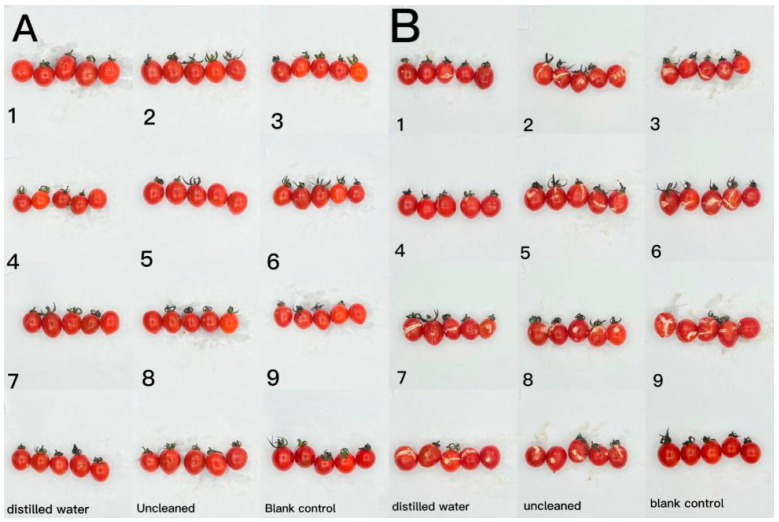
The effect of water-soluble chitosan (CTS)/curdlan (CUR) coatings on picked cherry tomatoes inoculated with *Botrytis cinerea* was studied. Photographs were taken on day 1 (**A**) and day 3 (**B**) after inoculation. In this figure, 1, 2, 3, 4, 5, 6, 7, 8, and 9 replaced CTS:CUR (*w*/*w*) = 1:0, 0:1, 1:0.5, 1:1, 1:1.5, 1:2, 0.5:1, 1.5:1, and 2:1, respectively.

**Figure 10:**
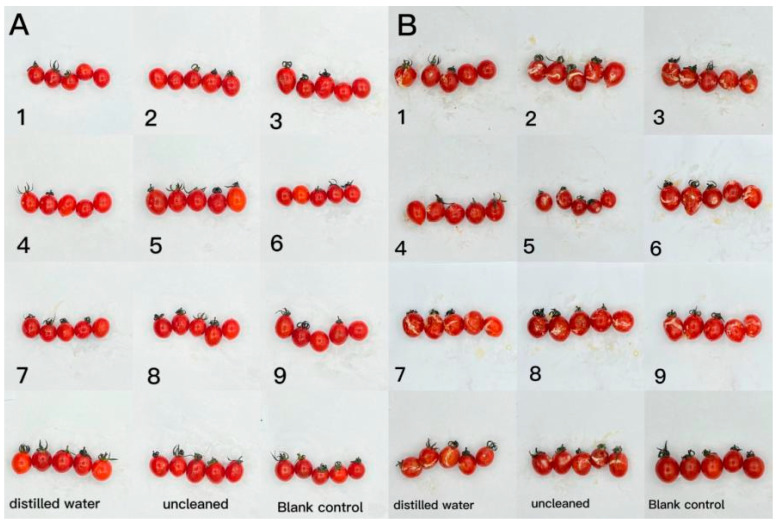
The effect of water-soluble chitosan (CTS)/curdlan (CUR) coatings on picked cherry tomatoes inoculated with *Alternaria* (Nees: Fr) was studied. Photographs were taken on day 1 (**A**) and day 3 (**B**) after inoculation. In this figure, 1, 2, 3, 4, 5, 6, 7, 8, and 9 replaced CTS:CUR (*w*/*w*) = 1:0, 0:1, 1:0.5, 1:1, 1:1.5, 1:2, 0.5:1, 1.5:1, and 2:1, respectively.

The effect of CTS/CUR composite coatings on the resistance of postharvest cherry tomatoes to *Botrytis cinerea* and *Alternaria* (Nees: Fr) was further investigated. Figure 9 and Figure 10 demonstrate that a pure coating of CTS had the most effective inhibitory effects on *Botrytis cinerea* and *Alternaria* (Nees: Fr). Furthermore, composite coating No. 4, with a CTS/CUR ratio of 1:1(*w*/*w*), exhibited excellent inhibitory effects on *Botrytis cinerea* and *Alternaria* (Nees: Fr) in vitro [13].

## 4. Discussion

Water-soluble chitosan is a natural alkaline polysaccharide that can effectively inhibit plant pathogenic fungi, reduce the need for antifungal agents, and lower production costs [24]. Somenath Das et al. [25] reported that chitosan effectively inhibits fungi in *C. sinensis* fruits, while Muhammad et al. [26] reported that a composite coating of water-soluble chitosan has a significant inhibitory effect on gray mold in sweet cherries. These findings are consistent with our own experiments. In our study, antifungal experiments revealed that the curdlan coating had weak direct inhibitory effects. Curdlan, a natural macromolecular compound, performed worse than the CTS/CUR composite films, which demonstrated stronger molecular interactions than the pure CTS and CUR films. The composite films also exhibited changes in functional groups, compared to those of the individual substances, suggesting a possible reaction between the two components. The performances of the composite films varied with different concentrations of CUR, as observed through SEM, which revealed increased fragility and decreased moisture resistance. These SEM results were consistent with the tensile strength, elongation at break, and moisture resistance test results. Notably, the in vitro validation experiment with cherry tomatoes yielded different conclusions than the preservation experiment. This phenomenon has been observed previously [13]. The results indicated that a CTS/CUR ratio of 1:1 exhibited remarkable antifungal activity (Figure 10) and deviated from previous findings. It is hypothesized that this difference may be due to the synergistic inhibition of fungi, although other reactions, such as enzyme activity in cherry tomatoes, cannot be ruled out. This assumption requires further confirmation through additional experiments. Although the exact mechanism of fungal inhibition by composite coatings on cherry tomatoes has not yet been fully elucidated, these results support the potential use of CTS/CUR composite coatings as an effective material for controlling postharvest fungal diseases in cherry tomatoes [27,28].

## 5. Conclusions

In this study, a novel edible composite coating was developed by incorporating CUR into a CTS coating solution. These findings revealed that the addition of CUR substantially enhanced the physical, mechanical, and barrier properties of the composite films. Compared to those of the pure CTS and CUR films, the composite films, specifically those with a 1:1 mass ratio of CTS to CUR, exhibited superior qualities. Furthermore, the CTS/CUR composite coating at a 1:1 ratio effectively preserved the postharvest quality of the cherry plants. Therefore, CTS/CUR edible composite coatings have the potential to significantly extend the shelf life of cherry tomatoes and could be applied in fruit and vegetable preservation in the future.

## Figures and Tables

**Table 1:** Results of thermogravimetric analysis of water-soluble chitosan (CTS)/curdlan (CUR) films.

Sample	T10% Degradation Temperature/°C	T25% Degradation Temperature/°C	T50% Degradation Temperature/°C	Mass Residue at 700 °C/%
1	138.708	200.972	290.487	12.937
2	261.611	361.418	\	58.961
3	151.598	203.406	315.679	17.150
4	133.71	236.638	399.585	39.333
5	84.4059	199.106	350.6	29.401
6	168.731	255.016	535.3	44.004
7	193.035	275.467	560.155	44.491
8	182.891	261.935	461.711	43.8033
9	147.193	215.677	322.162	23.474

T10%, T25%, and T50% represent the temperatures when the mass losses are 10%, 25%, and 50%, respectively. In this table, 1, 2, 3, 4, 5, 6, 7, 8, and 9 replaced CTS:CUR (*w*/*w*) = 1:0, 0:1, 1:0.5, 1:1, 1:1.5, 1:2, 0.5:1, 1.5:1, and 2:1, respectively.

**Table 2:** Results of the physical performance analysis of water-soluble chitosan (CTS)/curdlan (CUR) films.

Sample	MC/%	WS/%	Opacity/%
1	13.68 ± 0.06 ^a^	18.01 ± 0.01 ^b^	43.07 ± 0.03 ^a^
2	13.39 ± 0.13 ^a^	12.29 ± 0.01 ^d^	3.05 ± 0.02 ^d^
3	10.15 ± 0.03 ^d^	16.31 ± 0.07 ^c^	3.26 ± 0.05 ^d^
4	9.65 ± 0.13 ^e^	15.67 ± 0.06 ^b^	2.22 ± 0.03 ^e^
5	7.41 ± 0.10 ^f^	14.83 ± 0.04 ^c^	1.09 ± 0.04 ^f^
6	6.37 ± 0.13 ^g^	11.52 ± 1.28 ^d^	0.58 ± 0.06 ^f^
7	7.630 ± 0.23 ^f^	20.75 ± 0.04 ^a^	1.13 ± 0.03 ^g^
8	10.58 ± 0.22 ^c^	11.61 ± 0.02 ^d^	4.93 ± 0.02 ^c^
9	11.28 ± 0.06 ^b^	10.57 ± 0.02 ^d^	5.94 ± 0.10 ^b^

Means in each column with different superscript letters are significantly different (*p* ≤ 0.05). In this table, 1, 2, 3, 4, 5, 6, 7, 8, and 9 replaced CTS:CUR (*w*/*w*) = 1:0, 0:1, 1:0.5, 1:1, 1:1.5, 1:2, 0.5:1, 1.5:1, and 2:1, respectively.

**Table 3:** Results of mechanical properties analysis of water-soluble chitosan (CTS)/curdlan (CUR) films.

Sample	Thickness/mm	TS	EAB/%
1	0.338 ± 0.04 ^e^	2.96596 ± 0.01 ^e^	2.64 ± 0.07 ^i^
2	0.738 ± 0.01 ^a^	6.27588 ± 0.007 ^b^	20.90 ± 0.363 ^e^
3	0.23 ± 0.03 ^f^	7.91589 ± 0.03 ^a^	4.95 ± 0.08 ^h^
4	0.432 ± 0.02 ^d^	5.17245 ± 0.02 ^c^	7.40 ± 0.07 ^g^
5	0.542 ± 0.01 ^c^	4.44372 ± 0.07 ^d^	13.19 ± 0.07 ^f^
6	0.544 ± 0.01 ^c^	1.53531 ± 0.07 ^g^	25.58 ± 0.07 ^c^
7	0.438 ± 0.02 ^d^	1.47896 ± 0.07 ^g^	32.93 ± 0.07 ^a^
8	0.66 ± 0.01 ^b^	2.01635 ± 0.07 ^f^	28.14 ± 0.07 ^b^
9	0.628 ± 0.01 ^b^	2.95441 ± 0.07 ^e^	25.09 ± 0.07 ^d^

Means in each column with different superscript letters are significantly different (*p* ≤ 0.05). In this table, 1, 2, 3, 4, 5, 6, 7, 8, and 9 replaced CTS:CUR (*w*/*w*) = 1:0, 0:1, 1:0.5, 1:1, 1:1.5, 1:2, 0.5:1, 1.5:1, and 2:1, respectively.

**Table 4:** Results of barrier performance analysis of water-soluble chitosan (CTS)/curdlan (CUR) films.

Sample	Water Vapor Permeability g/(h·m^2^)	CDP cm^2^/(d·kPa)	PV (g/100 g)
1	0.090856481 ± 0.0007 ^a^	0.05875 ± 0.0004 ^a^	1.99 ± 0.2 ^c^
2	0.064252097 ± 0.0004 ^e^	0.00877 ± 0.0015 ^f^	0.45 ± 0.04 ^f^
3	0.059267648 ± 0.0007 ^f^	0.05374 ± 0.0006 ^a^	1.46 ± 0.10 ^d^
4	0.066328686 ± 0.0007 ^d^	0.02858 ± 0.0018 ^d^	1.68 ± 0.09 ^d^
5	0.066497247 ± 0.0007 ^d^	0.01273 ± 0.0008 ^e^	2.16 ± 0.12 ^c^
6	0.090074584 ± 0.0007 ^a^	0.01133 ± 0.001 ^e^	3.04 ± 0.09 ^a^
7	0.060466012 ± 0.0007 ^f^	0.02353 ± 0.0006 ^d^	2.73 ± 0.09 ^b^
8	0.079594423 ± 0.0007 ^b^	0.03875 ± 0.002 ^c^	1.47 ± 0.11 ^de^
9	0.076399027 ± 0.0007 ^c^	0.04042 ± 0.002 ^b^	1.26 ± 0.07 ^e^

Means in each column with different superscript letters are significantly different (*p* ≤ 0.05). In this table, 1, 2, 3, 4, 5, 6, 7, 8, and 9 replaced CTS:CUR (*w*/*w*) = 1:0, 0:1, 1:0.5, 1:1, 1:1.5, 1:2, 0.5:1, 1.5:1, and 2:1, respectively.

## Data Availability

The data presented in this study are available on request from the corresponding author.
